# NAVIGATING COMPLEXITY: EFFECTIVE PEDAGOGY FOR TEACHING DIFFICULT TOPICS

**DOI:** 10.1093/geroni/igae098.0884

**Published:** 2024-12-31

**Authors:** Cory Bolkan, Raven Weaver, Autumn Decker

**Affiliations:** Washington State University, Pullman, Washington, United States; Washington State University, Pullman, Washington, United States; Pacific University, Hillsboro, Oregon, United States

## Abstract

There are many sensitive (e.g., death, loss, trauma) and politically charged (e.g., aging policies, ageism) topics that often emerge in gerontology courses that can challenge educators and impede classroom dialogue. We surveyed undergraduate students (N = 380; 76% Women, 65% White, 44% first-year) regarding their perspectives on which topics they believed were important to feature in lifespan human development curricula. Results indicated strong interest in specifically addressing challenging issues in their classes, with the top, most-reported topics identified as mental illness (n = 252) and death and loss (n = 193). Additional topics of high interest included substance abuse, gun violence, discrimination, and maltreatment. Incorporating these topics into gerontological education offers many benefits and fosters comprehensive understanding of the full life course among students. To help educators navigate these topics effectively, we provide an overview of various pedagogical approaches that consider social crises and power relations (e.g., culturally responsive teaching, critical pedagogies, inquiry-based or experiential learning), and share examples of how they can be used to guide and facilitate learning about sensitive issues. Trauma-informed teaching also ensures a safe learning environment for discussing difficult topics, while critical pedagogies encourage critical analysis of societal norms and power dynamics to broaden students’ understanding. By integrating these approaches, educators can create inclusive learning environments where students are empowered to explore and discuss multifaceted issues relevant to later life. This enhances academic learning and equips students with the skills and empathy needed to address the challenges faced by a diverse population of older adults

